# Long noncoding RNA CEBPA-DT promotes cisplatin chemo-resistance through CEBPA/BCL2 mediated apoptosis in oral squamous cellular cancer

**DOI:** 10.7150/ijms.64253

**Published:** 2021-09-27

**Authors:** Xue Qiao, Jiayi Liu, Li Zhu, Rongbo Song, Ming Zhong, Yan Guo

**Affiliations:** 1 Department of Central Laboratory, School and Hospital of Stomatology, China Medical University, Liaoning Province Key Laboratory of Oral Disease.; 2 Department of Oral Biology, School and Hospital of Stomatology, China Medical University, Liaoning Province Key Laboratory of Oral Disease.; 3 Department of Oral Pathology, School and Hospital of Stomatology, China Medical University, Liaoning Province Key Laboratory of Oral Disease.; 4 Department of Stomatology, Xiang'an Hospital of Xiamen University, Xiamen, China.

**Keywords:** oral squamous cell carcinoma, cisplatin, CEBPA Divergent Transcript, CEBPA, apoptosis

## Abstract

Intrinsic or developing resistance to chemotherapy drugs including cisplatin (CDDP) remains the major limitation of cancer therapeutic efficacy in cancers. Recently, increasing evidence suggested that long noncoding RNAs (lncRNAs) play a critical role in various biological processes of tumors, and have been implicated in resistance to various drugs. However, the role of lncRNAs in cisplatin resistance is poorly understood. Here, we found that the expression of lncRNA CEBPA-DT/CEBPA/BCL2 was upregulated in cisplatin resistance OSCC cells (Cal27-CisR and HSC4-CisR) compared with their parental cells (Cal27 and HSC4). CEBPA-DT overexpression could upregulated both cytoplasmic and nuclear CEBPA expression. Down-regulation of CEBPA-DT enhances cisplatin sensitivity, facilitates cell apoptosis in cisplatin-resistant OSCC cells. In addition, we identified that CEBPA-DT regulates cisplatin chemosensitivity through CEBPA/BCL2-mediated cell apoptosis. Knockdown of CEBPA and BCL2 could alleviate the increasement of cisplatin resistance induced by CEBPA-DT overexpression. Our findings indicate that downregulation of lncRNA CEBPA-DT may be a potential therapy to overcome cisplatin resistance in OSCC.

## Introduction

Oral cancers include tumors of a number of different subtypes that arise within the oral cavity, with oral squamous cell carcinoma (OSCC) being the most common of these malignancies. OSCC incidence has risen in recent years, with over 274,000 newly diagnosed cases globally accounting for 80-90% of primarily oral cancers 1, 2. The prognosis of OSCC is poor, as this tumor type exhibits invasive growth patterns and high rates of metastasis and recurrence 3. While chemotherapeutic treatment can somewhat improve OSCC patient prognosis 4, the emergence of chemoresistance is a major cause of treatment failure 5, 6. It is thus essential that the mechanistic basis for OSCC chemotherapy drug resistance be clarified so as to facilitate the development of more efficacious treatments.

Long non-coding RNAs (lncRNAs) have recently been identified as key regulators of malignant processes wherein they can function as tumor suppressors or oncogenes 7, 8. Specific lncRNAs have been linked to the emergence of chemoresistance in OSCC tumors 9, 10. CEBPA Divergent Transcript (CEBPA-DT, also known as CEBPA-AS1) is a 2252 nucleotide lncRNA encoded on chromosome 19q13.11 11-13. To date, just three studies have explored the dysregulation and functional relevance of CEBPA-DT in cancers. In gastric cancer, the enrichment of CEBPA-DT in tumor tissues was suggested to offer value as a diagnostic biomarker associated with this cancer type 11. Consistent with this, a recent analysis of The Cancer Genome Atlas (TCGA) database suggested that a 4-lncRNA signature which included CEBPA-DT was able to predict patient prognosis 14.

In our previous study, we demonstrated that CEBPA-DT can suppress OSCC cell malignancy, thus serving as a valuable biomarker of OSCC prognosis 12. However, the functional relevance of CEBPA-DT to chemoresistance has yet to be defined. Bioinformatics analysis together with gene coding sequence analysis through online databases revealed that CEBPA-DT, has no overlapping with CEBPA (Fig. S1), may regulate it following a “lncRNA-mRNA” pattern. Herein, we explored the detail function and mechanisms whereby this lncRNA regulates OSCC cell cisplatin (CDDP) chemosensitivity.

## Methods

### Cell lines and culture

Human HSC4 and Cal27 OSCC cells were obtained from the Chinese Academy of Sciences (Shanghai, China) and cultured in DMEM medium (Hyclone, USA) containing 10% fetal bovine serum (Gibco, USA) 12. The CDDP-resistant HSC4-CisR and Cal27-CisR cells were prepared as in prior reports by culturing them under gradually increasing CDDP concentrations. Resistance was then maintained by culturing these cells in 0.5 μM CDDP (Sigma, USA), with this drug only being omitted for the 7 days prior to each experiment.

### qPCR

Trizol (Thermo Fisher Scientific, USA) was used to extract RNA from cells, after which a lncRNA First-Strand cDNA Synthesis Kit (Tiangen, China) was used for cDNA preparation. A lncRNA qPCR Detection Kit (Tiangen, China) was then used based on provided directions with the following primers: CEBPA-DT: 5'-GCTTCGTTTTCGGTCCAGA-3' (sense) and 5'-CCCTCCACAGGTGAATGCTAT-3' (antisense); CEBPA: 5'-TTTGCTCGGATACTTGCCA-3' (sense) and 5'-AAAGGAAAGGGAGTCTCAGACC-3' (antisense); BCL2: 5'- GATTGAAGACACCCCCTCGT-3' (sense) and 5'-CCGGTTATCGTACCCTGTTCT-3' (antisense); GAPDH: 5'-GGGAGCCAAAAGGGTCAT-3' (sense) and 5'- GAGTCCTTCCACGATACCAA -3' (antisense) 12. CEBPA-DT relative expression was assessed via the 2^-ΔΔCT^ method, with GAPDH being used for normalization.

### Constructions and Cell Transfection

Smart Silencers specific for CEBPA-DT (ss-CEBPA-DT) and corresponding controls (ss-NC) were obtained from Ribobio (Guangzhou, China). Overexpression was achieved by cloning the full CEBPA sequence into the pcDNA plasmid (pc-CEBPA) obtained from Genescript (Nanjing, China), with an empty plasmid serving as the negative control (pc-NC). Lipofectamine™ 3000 (Invitrogen, USA) was used to transfect cells in 12-well plates based on provided directions.

### Drug sensitivity analyses

HSC4-CisR and Cal27-CisR cells were treated with CDDP (0, 1, 2.5, 5, 7.5, 10, 12.5, 25, and 50 μM). Viability was assessed at 24 h post-treatment, after which half-maximal inhibitory concentration (IC50) values were calculated based upon dose-response curves.

### Apoptosis analyses

An Annexin V-FITC apoptosis detection kit (Biosea, China) and TUNEL assays (Roche, Switzerland) were used to gauge apoptotic cell death. For the Annexin V-FITC kit, 2×10^5^ cells were spun down for 5 min at 1000 ×g, after cells were resuspended in 500 µl binding buffer containing 5µl each of PI and Annexin V-FITC. Following a 10 min staining step, cells were assessed via flow cytometry (CantoⅡ, BD) and analyzed with Diva 8.0 (BD, USA). Apoptotic cells were those that were FITC-Annexin V positive/PI negative. For TUNEL staining, 5×10^4^ cells were fixed for 30 min using 4% paraformaldehyde, permeabilized with 0.3% Triton X-100, and stained for 1 h with TUNEL fluorescent labeling solution. Samples were then probed for 15 min with PI and analyzed via microscopy (Nikon, Japan).

### Nuclear and cytoplasmic protein extraction

Nuclear and cytoplasmic protein were isolated through Nuclear and Cytoplasmic Protein Extraction Kit (Beyotime, China) following manufacturer's instructions. Briefly, cells were washed with PBS for 3 times, harvested and centrifuged at 12,000 g for 10 min. The cell sediments were resuspended in 200 μl Buffer A containing 1 mM Phenylmethylsulfonyl fluoride (PMSF) per 20 μg protein and homogenized on ice for 10-15 mins. 10 μl Buffer B was added and the homogenate was centrifuged at the 12,000-16,000 g for 5 min. Cytoplasmic protein were collected in the supernatants. Next, the remaining sediments were resuspended in 50 μl nuclear protein extraction buffer and were transfered to the ice for 30 mins homogenization with every 2 mins vortex. Finally, the resulting supernatants containing nuclear fraction were collected after 12,000-16,000 g centrifugation for 10 mins.

### Western Blotting

Chilled lysis buffer supplemented with protease and phosphatase inhibitors (KEYGEN, China) was used to extract protein, after which a BCA assay (KEYGEN, China) was used to quantify protein levels based on provided directions. Lysates (30 μg) were separated via 10% SDS-PAGE and transferred to 0.45 μm PVDF membranes that were subsequently blocked for 2 h with 5% non-fat milk and then probed overnight with rabbit anti-CEBPA (1:500, Cat# YT0551, Immunoway) or mouse anti-BCL2 (1:500, Cat# YM3041, Immunoway) overnight at 4 °C. Blots were then probed for 2 h with secondary antibodies, and a Dual Color Infra-red Laser Imaging System (Gene, HK, China) was used to detect protein bands. ImageJ (National Institutes of Health, USA) was used for protein band analyses. GAPDH (1:1000, Proteintech) served as a normalization control.

### Ethical Approval

This study has been carried out with the approval of the Ethics Committees of School of Stomatology. All procedures performed in studies involving human participants were in accordance with the ethical standards of the institutional and/or national research committee and with the 1964 Helsinki declaration and its later amendments or comparable ethical standards.

### Statistical analysis

Graphpad Prism 5.0 (GraphPad Software, Inc., CA, USA) was used for all statistical analyses. Data are given as means ± SD and were analyzed via one-way ANOVAs and two-tailed Student's t-tests. Experiments were repeated at least thrice, and P < 0.05 was the significance threshold for this study.

## Results

### The upregulation of CEBPA-DT is associated with OSCC cisplatin resistance

**Figure 1:**
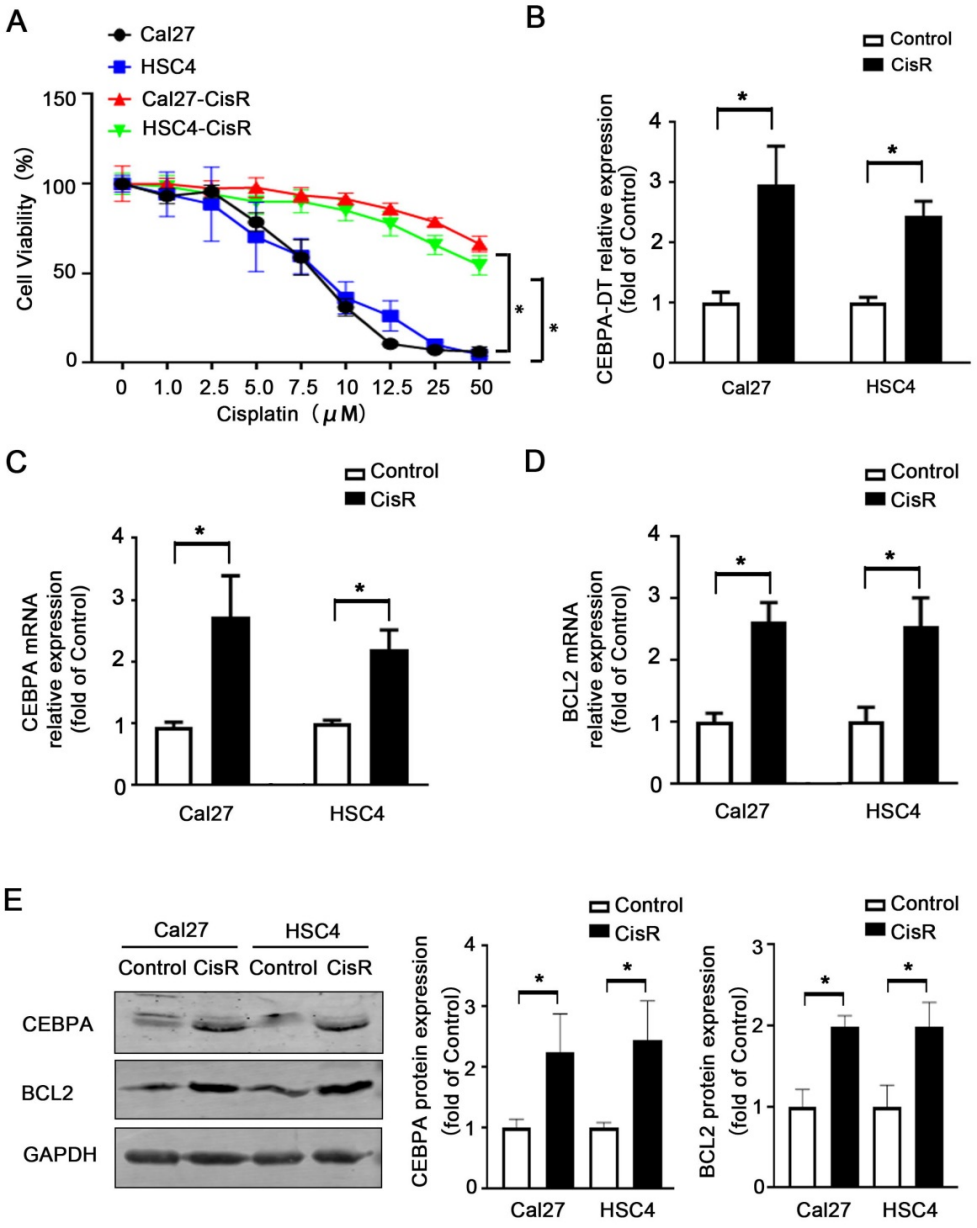
** CEBPA-DT up-regulation correlates to cisplatin resistance in OSCC.** (A) CCK-8 assay detected IC50 increase in chemo-resistance HSC4-CisR and Cal-CisR cells compared with normal parental cell lines. (B) qRT-PCR detected up-regulation of CEBPA-DT expression in chemo-resistance cell lines. (C-D) qRT-PCR detected mRNA relative expression of CEBPA and BCL2 in CisR cells relative to untreated control groups. (E) Western blot detected protein expression of CEBPA and BCL2 in CisR cells compared with control OSCC cell lines. N=3~4 independent experiments, *P < 0.05.

We have previously employed microarray and qPCR analyses to confirm that CEBPA-DT is upregulated in OSCC in a manner negatively correlated with tumor grading and clinical staging 12. Given that lncRNAs are known to mediate solid tumor resistance to different chemotherapeutic agents 15, 16, we next prepared chemoresistant strains of the HSC4 and Cal27 OSCC cell lines by treating them with continuous low doses of CDDP, yielding chemoresistant cells with higher IC50 values (Fig. 1A). We then assessed CEBPA-DT levels in these cells via qPCR, revealing significant upregulating of this lncRNA in both Cal27-CisR and HSC4-CisR cells relative to their parental cell lines (Fig. 1B). Moreover, CEBPA and BCL2 mRNA and protein expression levels were also dramatically upregulated in both CisR cell lines compared with control groups (Fig. 1C-E). These results indicated that the upregulation of CEBPA-DT/CEBPA/BCL2 was correlated with OSCC cell CDDP resistance.

### Downregulation of CEBPA-DT enhances OSCC cell sensitivity to cisplatin-induced apoptosis

**Figure 2:**
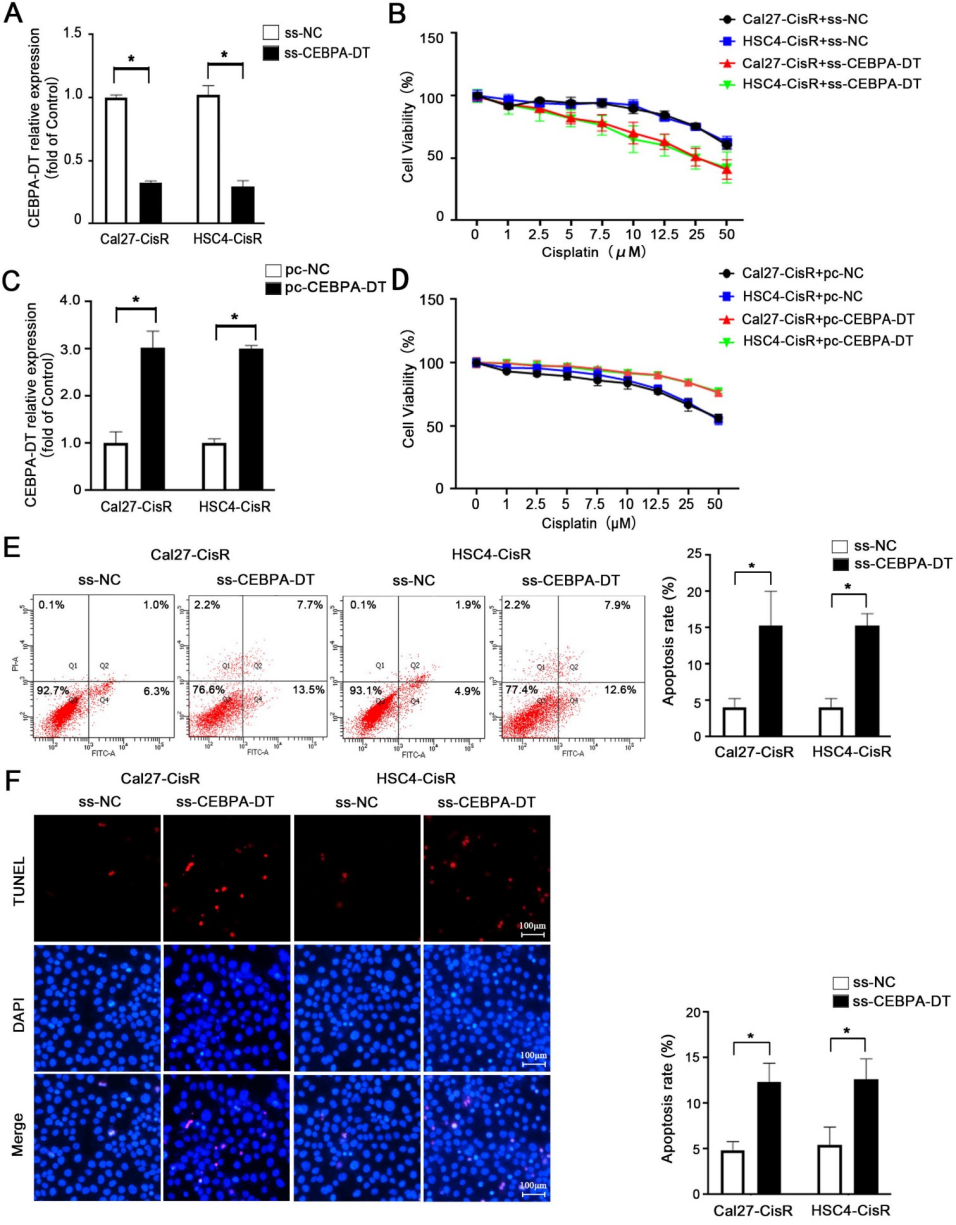
** CEBPA-DT downexpression enhances cisplatin sensitivity and facilitates cell apoptosis in cisplatin-resistant OSCC cells. (A)** qRT-PCR detected the transfection efficiency of CEBPA-DT smart silencer (ss-CEBPA-DT) and negative control (ss-NC) in Cal27-CisR and HSC4-CisR cells. **(B)** CCK-8 assay detected the IC50 value decrease in ss-CEBPA-DT transfected chemoresistance cells compared with ss-NC transfected group. **(C)** qRT-PCR detected CEBPA-DT overexpression (pc-CEBPA-DT) and corresponding control (pc-NC) transfection efficiency in Cal27-CisR and HSC4-CisR cells. **(D)** CCK-8 assay show IC50 increase in CEBPA-DT overexpression (pc-CEBPA-DT transfected) chemo-resistance cells compared with control (pc-NC transfected) group. **(E-F)** Cell apoptosis rate was detected in ss-CEBPA-DT and ss-NC transfected chemo-resistance cells through Annexin-V-FITC & PI staining assay (E) and TUNEL staining assay (F). N=3~5 independent experiments, *P < 0.05.

We next explored whether knocking down the expression of CEBPA-DT was sufficient to enhance the chemosensitivity of OSCC cells by transfecting them with smart silencer (ss-CEBPA-DT) or negative control (ss-NC) constructs (Fig. 2A). For gain-of-function experiments, this lncRNA was instead overexpressed in Cal27-CisR and HSC4-CisR cells by transfecting them with the pc-CEBPA-DT (pcDNA3.1-CEBPA-DT) or pc-NC vectors (Fig. 2C). A subsequent CCK-8 assay revealed that the knockdown of CEBPA-DT was sufficient to impair Cal27-CisR and HSC4-CisR cell viability, reducing their CDDP IC50 values to 13.36±1.73 μM and 11.81±0.39 μM, which were lower than those values for ss-NC cells (26.01±1.81 μM and 23.24±2.49 μM) (Fig. 2B). The overexpression of CEBPA-DT, in contrast, enhanced the survival of both tested CDDP-resistant OSCC cell lines relative to controls (Fig. 2D). The apoptotic death of these cells was then evaluated by flow cytometry, revealing that ss-CEBPA-DT transfection was associated with enhanced apoptotic death relative to ss-NC transfection even in the absence of CDDP treatment (data not shown). Upon such treatment, 4.06 ± 0.55% of Cal27-CisR and 4.10 ± 0.56% of HSC4-CisR ss-NC transfected cells exhibited signs of early apoptotic death, whereas these percentages rose to 15.34 ± 2.11% and 15.28 ± 0.75%, respectively, following CEBPA-DT knockdown (Fig. 2E). TUNEL staining further confirmed that the frequency of apoptotic cells was significantly higher in the CDDP-treated ss-CEBPA-DT group relative to the control group for both tested cell lines (Fig. 2F). Together, these results suggested that the downregulation of CEBPA-DT can enhance the sensitivity of OSCC cells to cisplatin.

### CEBPA-DT targets CEBPA to control cisplatin chemosensitivity in OSCC cells

**Figure 3:**
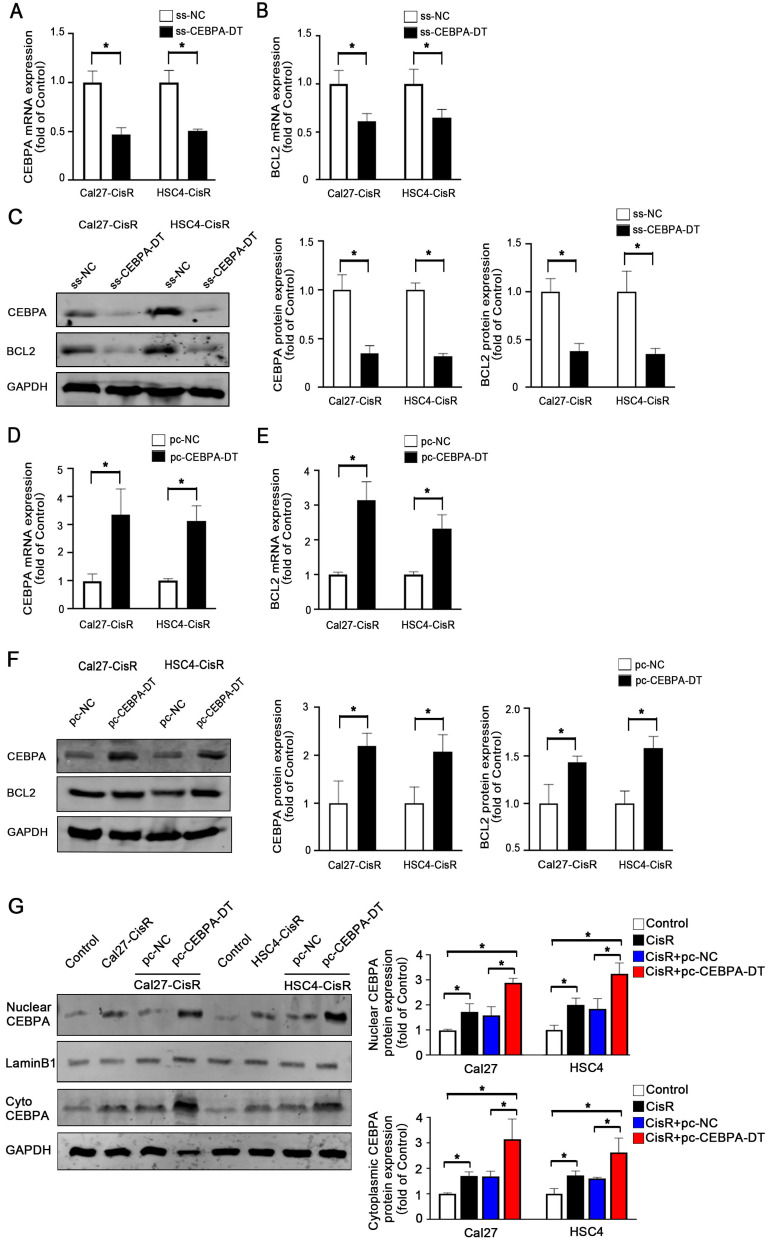
** CEBPA is the potential target of CEBPA-DT in regulating OSCC cisplatin chemosensitivity. (A-B)** qRT-PCR detected the CEBPA (A) and BCL2 (B) mRNA expression among ss-CEBPA-DT and ss-NC transfected Cal27-CisR and HSC4-CisR cells. **(C)** Western blot detected CEBPA and BCL2 protein expression in ss-CEBPA-DT and ss-NC transfected chemo-resistance cells. **(D-E)** qRT-PCR detected the CEBPA (D) and BCL2 (E) mRNA expression among pc-CEBPA-DT and pc-NC transfected Cal27-CisR and HSC4-CisR cells. **(F)** Western blot detected CEBPA and BCL2 protein expression in pc-CEBPA-DT and pc-NC transfected chemo-resistance cells. GAPDH was used as reference control. **(G)** Western blot detected CEBPA expression in cytoplasmic and nuclear protein separated in CisR and control cells, pc-NC and pc-CEBPA-DT transfected CisR cell lines, respectively. N=3 independent experiments, *P < 0.05.

CEBPA is a transcription factor that regulates the proliferation, differentiation, and apoptotic death of cells in a range of tissue types. Our above data demonstrating the importance of lncRNA CEBPA-DT in CDDP-resistant OSCC cells, we hypothesized that CEBPA-DT regulated cisplatin chemoresistance by targeting the downstream CEBPA/BCL2 signaling pathway. To test this, we evaluated the expression of CEBPA and BCL2 in CDDP-resistant OSCC cells in which CEBPA-DT had been knocked down or overexpressed via qPCR and Western blotting. This revealed that overexpressing CEBPA-DT significantly enhanced the expression of both of these genes at the mRNA and protein levels in both HSC4-CisR and Cal27-CisR cells (Fig. 3A-C). Likewise, knocking down CEBPA-DT inhibited CEBPA and BCL2 mRNA and protein expression in both tested cell lines (Fig. 3D-F). Moreover, we separated the nuclei and cytoplasmic protein and used western blot to detect the downstream CEBPA protein expression levels. The results showed that both the cytoplasmic and the nuclear CEBPA were up-regulated in CisR or pc-CEBPA-DT transfected CisR cells compared with control or pc-NC CisR cells (Fig. 3G). Together with their position relationship and the previous pull-down assay results that CEBPA is a target of CEBPA-DT in OSCC cells. Our data confirmed the ability of CEBPA-DT to regulate CEBPA/BCL2 expression in CDDP-resistant OSCC cells may be by a “lncRNA-mRNA” pattern, suggesting that CEBPA may be a key target of this lncRNA in the context of tumor chemoresistance.

### CEBPA-DT regulates CEBPA/BCL2-associated apoptosis to control OSCC cell chemosensitivity

**Figure 4:**
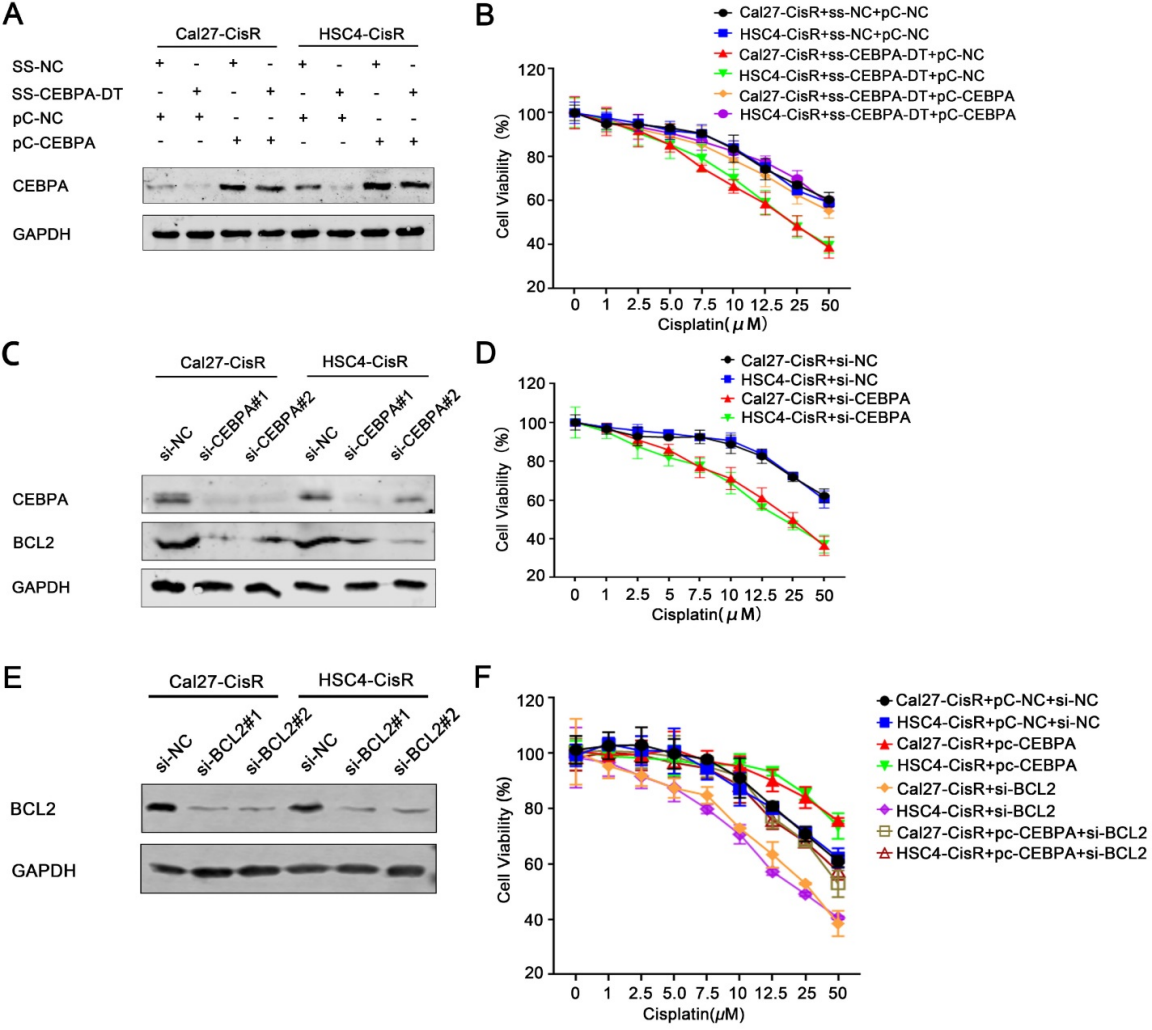
** CEBPA-DT regulates cisplatin chemosensitivity through CEBPA/BCL2 signaling pathway. (A)** qRT-PCR detected the transfection efficiency of CEBPA overexpression (pc-CEBPA) and corresponding negative control (pc-NC) in ss-CEBPA-DT/ss-NC co-transfected Cal27-CisR and HSC4-CisR cells. **(B)** CCK-8 assay detected the IC50 value in ss-CEBPA-DT and pc-CEBPA co-transfected chemo-resistance cells. **(C)** Western blot detected CEBPA and BCL2 protein expression in si-CEBPA and si-NC transfected chemo-resistance cells. **(D)** CCK-8 assay detected the IC50 value in si-CEBPA and si-NC transfected chemo-resistance cells. **(E)** Western blot detected BCL2 protein expression in si-BCL2 and si-NC transfected chemo-resistance cells. **(F)** CCK-8 assay detected the IC50 value in only si-BCL2 transfected, si-BCL2 and pc-CEBPA co-transfected chemo-resistance cells.

To explore the role of the CEBPA/BCL2 axis in the context of CEBPA-DT-mediated OSCC cell chemoresistance, we next overexpressed this transcription factor in OSCC cells transfected with ss-CEBPA-DT (Fig. 4A). Subsequent CCK-8 assays revealed that the overexpression of CEBPA was sufficient to enhance the viability and reduce the CDDP sensitivity of ss-CEBPA-DT co-transfected CDDP-resistant OSCC cells relative to cells transfected with the control pc-NC overexpression construct, increasing IC50 values from 13.47±0.40 μM and 11.86±0.36 μM to 21.26±0.41 μM and 19.53±0.47 μM (Fig. 4B). We then knocked down CEBPA in these tumor cell lines (Fig. 4C), revealing that this impaired the viability of Cal27-CisR and HSC4-CisR cells (Fig. 4D). Such CEBPA downregulation also significantly impaired BCL2 expression relative to control si-NC transfection in both tested cell lines (Fig. 4C). Moreover, we down-regulated BCL2 expression with siRNA transfection (Fig. 4E) following detected the changes of chemo-sensitivity. The results showed that the IC50 was dramatically decreased in CisR cells co-transfected with pc-CEBPA and BCL2 siRNA relative to those of pc-CEBPA transfected cells, which indicated that CEBPA overexpression induced chemo-sensitivity reduction was greatly rescued by BCL2 knockdown (Fig. 4F).

**Figure 5:**
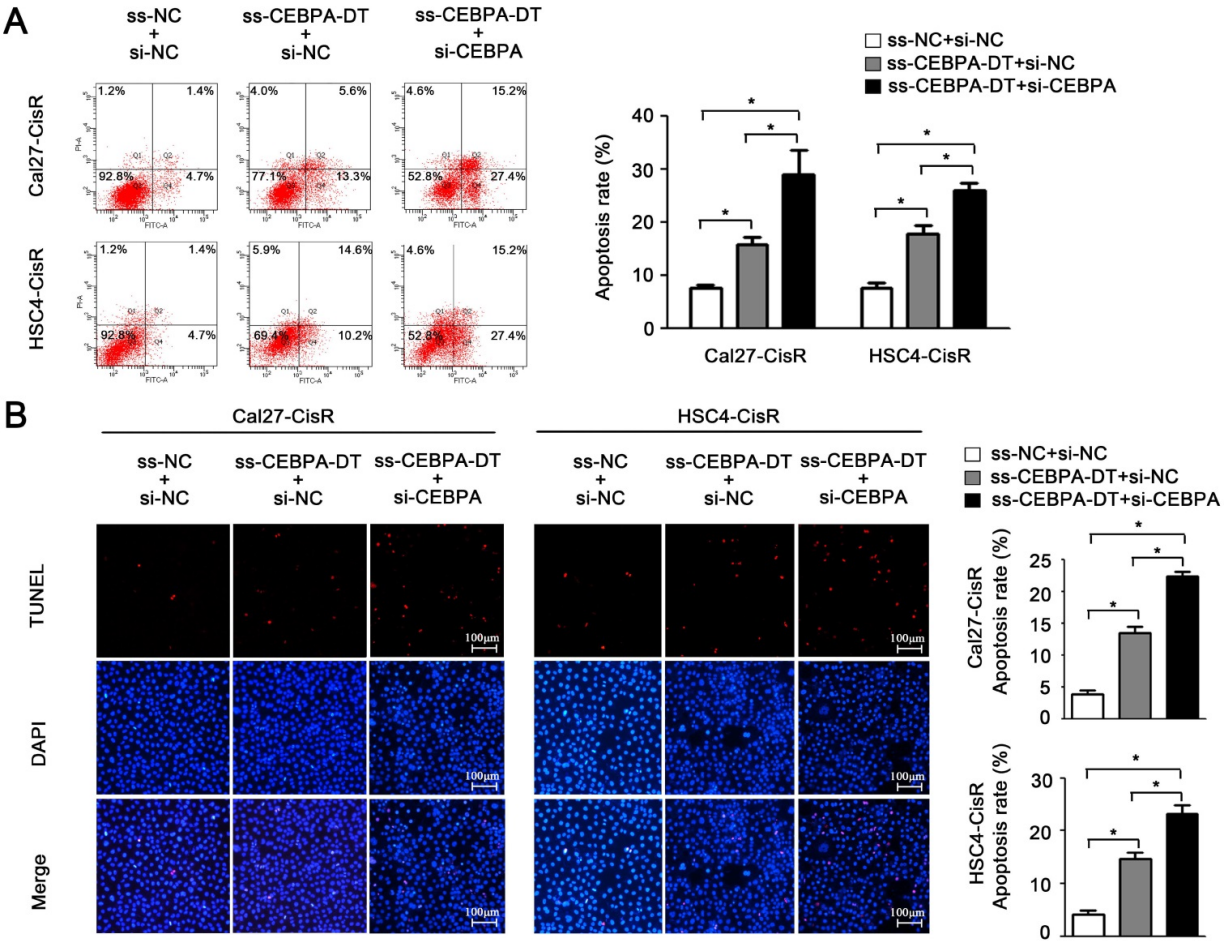
** CEBPA-DT regulates apoptosis through CEBPA in chemo-resistance cells. (A-B)** Cell apoptosis rate was detected in ss-CEBPA-DT/ss-NC and si-CEBPA/si-NC co-transfected chemo-resistance cells through Annexin-V-FITC & PI staining assay (A) and TUNEL staining assay (B). N=3~5 independent experiments, *P < 0.05.

And then, flow cytometry and TUNEL assays further confirmed that the downregulation of CEBPA enhanced apoptotic cell death in ss-CEBPA-DT co-transfected CDDP-resistant cells (Fig. 5A-B). Overall, these results suggested that CEBPA-DT is a lncRNA that can regulate the chemosensitivity of OSCC cells by regulating the CEBPA/BCL2 axis.

## Discussion

Cisplatin is a first-line chemotherapeutic agent that is used to treat many cancer types including OSCC. As a cytosine analog, CDDP can interfere with nucleic acid synthesis, resulting in chain termination and subsequent tumor cell death 17-19. The emergence of CDDP resistance, however, remains a major treatment barrier associated with negative patient outcomes.

Recent work has highlighted the importance of lncRNAs as mediators of the development and maintenance of CDDP resistance in OSCC cells. For example, knocking down the expression of the lncRNA UCA1 can significantly enhance tongue squamous cell carcinoma apoptosis in response to CDDP treatment through a mechanism linked to the inhibition of CDDP-induced PI3K/Akt signaling 20. Similarly, inhibiting expression of the lncRNA HOTAIR can disrupt OSCC cell autophagy to enhance CDDP chemosensitivity and associated apoptosis 21. UCA1 can promote OSCC cell malignancy in part by binding to miR-184 to indirectly regulate SF1 expression 22. We have previously identified CEBPA-DT as an oncogenic lncRNA associated with OSCC cell malignant phenotypes 12, revealing it to be upregulated in CDDP-resistant OSCC cells and suggesting it may thus play a role in the development of chemoresistance.

To confirm the importance of CEBPA-DT as a regulator of OSCC cell chemoresistance, we knocked down this lncRNA in two CDDP-resistant OSCC cell lines. Such knockdown reduced the CDDP IC50 value for these cells and enhanced their sensitivity to apoptotic death upon exposure to this chemotherapeutic drug. LncRNA were reported to have the potential of coding small peptide 23, which might regulate its nearby gene CEBPA-DT. We evaluated this ability through online bioinformatics tools (CPC2^24^ and PORTRAIT 25) and found that the coding potential of CEBPA-DT were 7.53% and 5.84% respectively, which suggested that there was almost no possibility that CEBPA-DT regulated CEBPA in a small peptide manner. Current opinions suggested that CEBPA-DT bound miRNA to regulate target gene 27, 28. Together with the position relationship of CEBPA-DT/CEBPA and our previous report that CEBPA is a target of CEBPA-DT, we thought that CEBPA-DT might promote cisplatin chemo-resistance by regulating CEBPA directly, which subsequently upregulate BCL2 expression in OSCC cells.

BCL2 localizes to the outer mitochondrial membrane wherein it can inhibit pro-apoptotic protein activity to enhance cell survival. Altered BCL2 expression or activity has been linked to chemoresistance in a range of cancer types. For example, miR-125b-5p can enhance gallbladder cancer cell sensitivity to chemotherapy by downregulating BCL2 expression 26, while CDKN2B-AS1 modulates the miR-125a-5p/BCL2 axis in endometrial carcinoma to enhance paclitaxel sensitivity 27. We thus hypothesized that CEBPA-DT was associated with OSCC cell resistance to CDDP through the modulation of the CEBPA/BCL2 pathway. To confirm this model, we evaluated the impacts of CEBPA-DT knockdown and confirmed that such knockdown impaired CEBPA and BCL2 expression in our CDDP-resistant OSCC cells. Subsequent rescue experiments revealed that CEBPA upregulation largely reversed the chemosensitivity-related effects of CEBPA-DT knockdown in these cells, thus confirming that CEBPA-DT can promote OSCC cell chemoresistance via the CEBPA/BCL2 signaling axis. However, there was some controversial comments on CEBPA function in solid cancers. Lourenço AR et al. suggested that CEBPA act as a tumor suppressor in solid tumors and a molecule for up-regulating CEBPA in a phase 1 clinical trial for liver cancer 28. Lu GD et al. suggested that CEBPA upregualtion is positively associated with tumor progression and classification 29-31. Our results showed that down-regulated CEBPA-DT, leading to downstream CEBPA and BCL2 deduction, might be a therapeutic approach for overcoming cisplatin resistance, which indicated CEBPA act as an oncogene in OSCC.

In summary, cisplatin-resistant OSCC cells exhibited CEBPA-DT upregulation, and knocking down this lncRNA enhanced the CDDP chemosensitivity of these cells via modulating the CEBPA/BCL2 pathway in OSCC. These data offer insight regarding the mechanisms governing OSCC chemoresistance, and further research will enable the appropriate selection of novel therapeutic targets associated with this cancer type.

## Supplementary Material

Supplementary figure.
