# Supplementary figures and images for: Long noncoding RNA CEBPA-DT promotes cisplatin chemo-resistance through CEBPA/BCL2 mediated apoptosis in oral squamous cellular cancer
Source: Int J Med Sci. 2021 Sep 27;18(16):3728–37. doi: 10.7150/ijms.64253 (PMC8579301; doi:10.7150/ijms.64253)

Supplemental Data

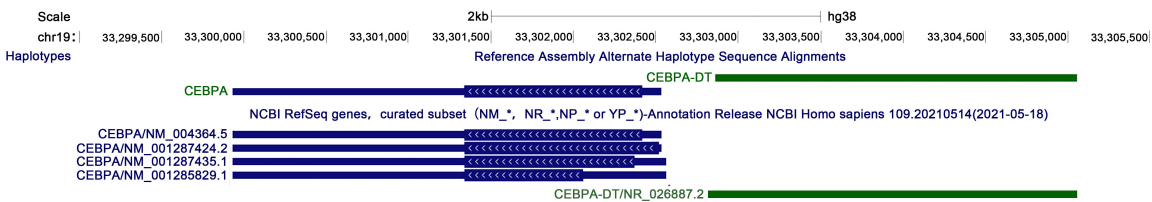

Figure S1

Supplement: Supplementary file 1 — Supplementary figure. [file ijmsv18p3728s1.pdf]
